# A brief novel intervention for acrophobia (fear of heights)

**DOI:** 10.1192/bjo.2021.351

**Published:** 2021-06-18

**Authors:** E. Naomi Smith

**Affiliations:** South London and Maudsley Training Scheme

## Abstract

**Objective:**

To investigate a unique brief intervention, which offers a combination of neuro-linguistic programming and practical graded exposure therapy, to overcome a fear of heights.

**Background:**

A fear of heights or acrophobia is common and often deters people from perusing activities like climbing. It can also interfere with routine activities of daily living.

**Case report:**

This two-day intervention is set in the Peak District (Derbyshire, UK) and works with a maximum of eight individuals to four instructors. The first half-day involves working with a psychotherapist using neurolinguistic programing techniques. The next 1.5 days involves graded exposure using abseiling over gradually increasing heights, to a final height of approximately 40 feet.

**Discussion:**

All eight individuals on the two-day course felt their fear of highs had significantly decreased. All eight individuals would recommend this intervention to others suffering from a fear of heights.

**Conclusion:**

It is noteworthy that the group undergoing this intervention were self-selected and highly motivated to overcome their fear of heights. The sample size was small and outcome measures were subjective. However, this is a novel and effective approach to helping people overcome their fear of heights. Further research with larger sample sizes would be beneficial in further assessing the impact of this intervention.

**Declaration:** Permission was granted by the organizers of this intervention to submit an abstract to conference. There are no conflicts of interests. This intervention is run by a private company ‘Will4Adventure’, I have no finical or other interests in this company. I privately funded my own place on this course.

